# Polariton probing of attometre displacement and nanoscale strain in ultrashort acoustic pulses

**DOI:** 10.1038/s41563-025-02229-3

**Published:** 2025-05-15

**Authors:** Marek Karzel, Anton K. Samusev, Tetiana L. Linnik, Mario Littmann, Dirk Reuter, Manfred Bayer, Andrey V. Akimov, Alexey V. Scherbakov

**Affiliations:** 1 Experimentelle Physik 2, Technische Universität Dortmund, Dortmund, Germany; 2 Department of Theoretical Physics, V. E. Lashkaryov Institute of Semiconductor Physics, Kyiv, Ukraine; 3 Department Physik, Universität Paderborn, Paderborn, Germany; 4 School of Physics and Astronomy, University of Nottingham, Nottingham, UK

**Keywords:** Imaging and sensing, Semiconductors, Optomechanics, Ultrafast photonics

## Abstract

Atomic displacement and lattice strain are inextricably linked to most ultrafast processes in solids, such as optically induced phase transitions or demagnetization. Visualizing lattice dynamics, which is typically done using time-resolved X-ray and electron diffraction techniques, yields information about the physical processes involved. However, the detection of atomic motion of an amplitude much less than a picometre has remained challenging. For this purpose, we suggest exploiting the acoustic pulse generated by a spatially localized ultrafast process in the surrounding volume. Its optical detection in a material possessing a narrow polariton resonance provides superior sensitivity. In the validating experiment, we detect the acoustic pulse generated by a 100 attometre thermal expansion of a 100 nanometre metallic film heated with a temperature increase of 0.2 kelvin by a femtosecond optical pulse. Even though the generated acoustic pulse carries dynamical strain with a magnitude of only 10^−9^, being injected into the polaritonic layer, it can be confidently detected through transient reflectivity.

## Abstract (web summary)

Polariton probing of high-frequency acoustic pulses enables the detection of coherent acoustic phonons, allowing the authors to monitor atomic motions in the 100 attometre range relative to their lattice position.

## Main

Ultrafast classical and quantum processes in nanoobjects are usually associated with the generation of stress, followed by the appearance of strain and the corresponding motion of atoms. Prominent examples of processes of this kind are the photoinduced structural phase transitions accompanied by a change of the elementary cell symmetry and the period of the crystalline lattice^1–7^, the generation of thermal stress due to electron gas and lattice heating in optically excited metal nanolayers^8,9^ and the ultrafast stress due to the deformation potential that follows the optical excitation of a semiconductor nanostructure, for example, a quantum dot, a quantum well or a superlattice^10–12^. The lattice response also accompanies the optically induced modification of magnetic order in ferromagnets^13–17^ and antiferromagnets^18^ and the changes of polarization in ferroelectrics^19–22^. All mentioned effects are also observed in the plethora of van der Waals materials actively studied nowadays^23–25^. Observing lattice kinetics in the time domain reveals the mechanisms involved in an investigated ultrafast process.

Ultrafast X-ray^1,2,7,9,10,13–15,17,19,20,22,26^ and electron^3,6,8,13,16,18,23–25,27^ diffraction are the most developed tools for directly observing atomic motion. Typically, the detectable displacement of the atomic planes and the associated strain in pump–probe X-ray and electron diffraction experiments are on the order of a picometre and 10^−4^, respectively^1–3,6–10,13–20,22–25^. These values are adequate for observing the lattice rearrangement in an ultrafast phase transition or the lattice expansion due to laser-induced heating by ~10 K and higher. However, in some cases, this sensitivity is insufficient. An actual example is optically induced spin dynamics in conventional^28^ and non-collinear^29^ antiferromagnets, in which the role of the lattice rearrangement in the spin reorientation transition is still unclear. However, the lattice strain induced by a complete orientational transition in these materials is ~10^−6^ (refs. ^30,31^), which makes its transients undetectable for ultrafast X-ray and electron diffraction techniques.

As an alternative to directly detect the motion of atoms of a nanostructure, we suggest focusing on the coherent perturbation induced by this process in the surrounding volume^4,11,14,17,32^. In the case of elastic contact with a solid environment (for example, a nanolayer on a substrate, a quantum dot in a bulk crystal and so on), the local atom movement generates a coherent acoustic pulse that propagates away from the object in the solid medium and can be measured distantly without any influence on the initial transient process. Such type of measurement exploiting the optical detection of acoustic waves has become an attractive tool in modern technologies, including quantum manipulation^33,34^, sensing^35,36^ and bioimaging^37^.

Numerous optical methods exist for detecting acoustic waves with frequencies from the gigahertz up to the terahertz range^38^. For instance, interferometric monitoring of the light reflected from the sample surface allows the detection of the surface displacement induced by a propagating acoustic pulse on the scale of 10^−14^ m with picosecond time resolution^39^, while the smallest detectable dynamical strain amplitude does not fall below 10^−7^. These values surpass the sensitivity of ultrafast X-ray diffraction. However, they are still too large to monitor the lattice dynamics accompanying spin reorientation in antiferromagnets^31^ or elastic imaging of living cells^37^, where the external influence should be critically small. These limitations motivate the search for more sensitive tools for detecting high-frequency acoustic waves. An increase in the sensitivity for detecting the injected acoustic wave by several orders of magnitude is also in great demand for prospective high-frequency quantum phononics operating with single acoustic quanta^40,41^.

## Polariton probing concept

The basic mechanism for the optical detection of high-frequency acoustic waves is the photoelastic effect, where the dynamical strain, *η*(*z*,*t*), associated with the acoustic wave induces optical reflectivity changes proportional to d*k*/d*η*, where *k* is the light wave vector in the medium, *z* is spatial coordinate along the propagation direction and *t* is time^42^. In materials without optical resonances, this coefficient is about the inverse wavelength of the probe light^42^, and for state-of-the-art experimental techniques^43^, the detectable strain amplitude, *η*_0_, carried by an acoustic wave is *η*_0_ ≈ 10^−6^. The strength of the photoelastic effect substantially increases for photon energies, *E* = *ħω* (ℏ is the reduced Plank constant and *ω* is the angular photon frequency) in the vicinity of an optical resonance, whose energy *E*_0_ shifts with the strain due to the deformation potential effect^44^. Then

1 $$\frac{{{\mathrm{d}}k}}{{\mathrm{d}}\eta }=\frac{1}{2}\frac{k}{\varepsilon }\frac{{\mathrm{d}}\varepsilon }{{\mathrm{d}}{E}_{0}}{\varXi },$$

where *ε* is the dielectric function of the medium with the optical resonance and *Ξ* is the deformation potential. It is clear from equation (1) that for reaching a high sensitivity, a large value of *Ξ* together with a spectrally narrow resonance is required, which results in a large slope d*ε*/d*E*_0_. Concrete examples comprising such a combination are systems hosting polaritons as strongly coupled states of excitons and photons^45^.

**Fig. 1:**
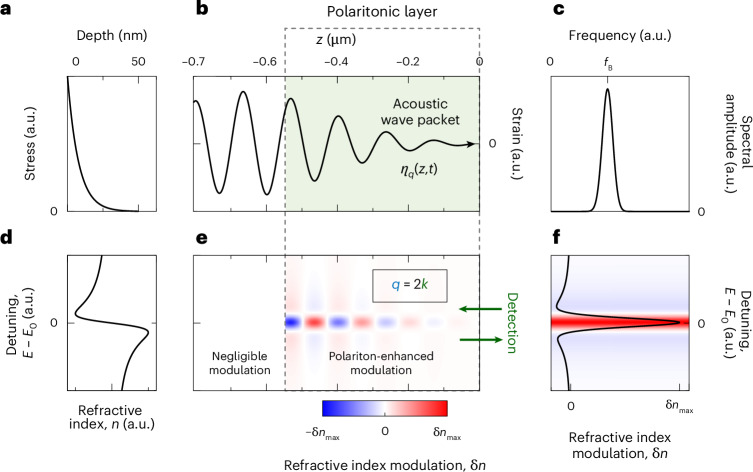
Polaritonic probing of acoustic pulse. **a**, Illustrative spatial profile of the stress induced as result of ultrafast optical excitation. **b**,**c**, Spatial profile *η*_*q*_(*z*,*t*) of the spectral component of the acoustic pulse detected through polaritonic probing (**b**). Its fast Fourier transform shown in **c** consists of a single spectral line, which is centred at the frequency *f*_B_, determined by fulfilling the Brillouin scattering condition, and has a spectral width determined by the absorption length of the probe light in the polaritonic layer. **d**, Dispersion of the refractive index (real part) in spectral vicinity of the polariton resonance as function of the detuning between the probe photon energy, *E*, and the polariton resonance energy, *E*_0_. **e**, Spatio-spectral representation of the refractive index modulation in the polaritonic layer induced by the *η*_*q*_(*z*,*t*) harmonic of the acoustic pulse shown in **b**. The spatial modulation period corresponds to half of the optical probe wavelength in the polaritonic layer. The maximum refractive index modulation δ*n*_max_ is achieved at the point of maximum strain amplitude when it is situated inside the polaritonic layer at zero detuning between the probe photon and the polariton resonance energies, as illustrated in **f**. **f**, Strain-induced modulation of the refractive index when achieving its maximum value as a function of the detuning between the probe photon and the polariton resonance energies. The coloured shading corresponds to the colour bar in **e**. Source data

Figure 1 schematically shows a hypothetical experiment where polariton probing is exploited for the detection of an acoustic pulse emitted into a solid environment as a result of some ultrafast transient process. The stress generated in a nanometre-thick layer (Fig. 1a) injects an acoustic pulse *η*(*z*,*t*), which propagates along the *z* axis with the sound velocity *v*. The pulse reaches a polaritonic layer (Fig. 1b) featuring a narrow optical resonance and playing the role of an acoustic pulse detector. The dynamical strain *η*(*z*,*t*) of the acoustic wave packet (Fig. 1b) modulates the optical properties of the polaritonic layer due to the photoelastic effect (Fig. 1e) and, thus, the intensity of the reflected optical probe beam. For a given polariton resonance dispersion of the real part of the refractive index, $n(E)=\mathrm{Re}\sqrt{\varepsilon \left(E\right)}$, as illustrated in Fig. 1d, the reflectivity change $\Delta R\left(t\right)=R\left(t\right)-{R}_{0}$ (with *R*_0_ being the reflectivity without the acoustic wave) is maximal when *E* is tuned exactly to the polariton resonance, *E*_0_. A typical dependence of the strain-induced refractive index modulation on the probe photon energy is shown in Fig. 1f. For the limiting case of an infinite polaritonic layer, for normal incidence of the probe light, and for studying a non-absorbing material, a strict selection rule holds for the relation between the acoustic and optical momenta: *q* = 2*k* (with *q* representing the amplitude of the phonon wave vector). The spatial modulation of the refractive index by the corresponding harmonic of the acoustic pulse, *η*_*q*_(*z*,*t*), has a period of half of the probe wavelength, as illustrated in Fig. 1e, forming the dynamical Bragg reflector for the probe beam. The layer’s high reflectance is provided by the high refractive index contrast due to the strong modulation of *n*(*E*) in the spectral vicinity of the polariton resonance. The resulting optical response Δ*R*(*t*) is a function oscillating with the following frequency^46^:

2 $${f}_{{\mathrm{B}}}=\frac{2v\sqrt{\varepsilon }}{{\lambda }_{0}},$$

where *λ*_0_ is the probe wavelength in vacuum. The temporal modulation of the reflected probe light is due to the interference of the probe beam reflected at the open surface and at the acoustic pulse. This effect is often called ‘time-domain Brillouin scattering’^47^ due to the mentioned selection rule (*f*_B_ = *vq*/2π; *q* = 2*k*), which is the same as that of spontaneous Brillouin light scattering. Note that for a real polaritonic layer of finite size and in the presence of absorption, the acoustic wave packet harmonic *η*_*q*_(*z*,*t*) detected by the set-up (Fig. 1b) has a finite duration and thus a finite spectral width with the spectral maximum given by equation (2) (Fig. 1c). The spatio-spectral profile of the refractive index modulation within the polaritonic layer shown in Fig. 1e illustrates the case of such a finite acoustic wave packet.

The polariton resonance in the hypothetical experiment described above provides an extremely high sensitivity for acoustic pulse detection in comparison with standard experiments without polariton resonance. Although the role of polaritons in the photoelastic effect has been widely discussed by now (a review is in the literature^48^), the limits of the polariton detection sensitivity for extremely small values of strain and displacement associated with high-frequency acoustic waves have not been explored. In this Article we show that by combining spatially extended detection of the acoustic pulse propagating in a medium featuring a polariton resonance with a detection technique based on dynamical interference, we can achieve a sensitivity well beyond those achievable with other techniques.

## Polariton probing of thermal expansion of a metal film

**Fig. 2:**
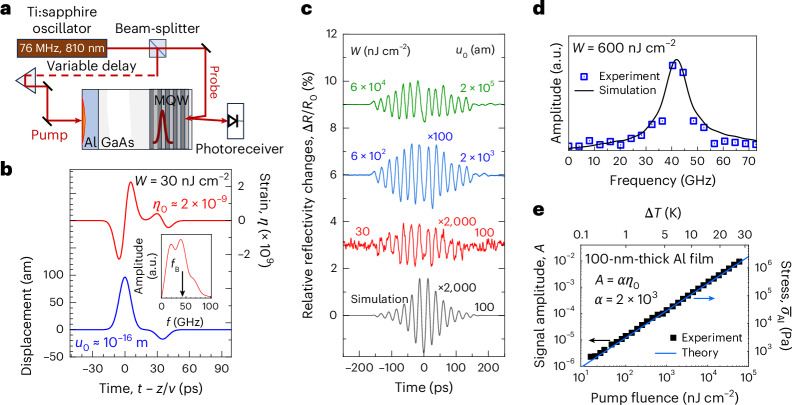
Detection of attometre displacement generated by optical heating of the electron gas. **a**, Experimental scheme. **b**, Temporal profile of the acoustic pulse injected into the polaritonic layer: displacement *u*(*t* – *z*/*v*) (blue line) and strain *η*(*t* – *z*/*v*) (red line). The inset shows the spectrum of the acoustic pulse obtained by fast Fourier transform of the temporal strain profile. **c**, Measured and simulated transient reflectivity signals, Δ*R*(*t*)/*R*_0_. The three upper curves show the experimentally measured signals for three values of the pump fluence, *W*. The lower curve is calculated for the displacement amplitude of 100 am, which corresponds to *W* = 30 nJ cm^−2^. **d**, Normalized fast Fourier transform spectra of the experimental (*W* = 600 nJ cm^−2^) and calculated (*u*_0_ = 2,000 am) transient reflectivity signals calculated for the first half of the transient signal in the time interval of [–600 ps; 0 ps]. **e**, Measured and calculated dependences of the transient reflectivity signal amplitude (left axis) and the optically generated stress in the Al film (right axis) on the pump fluence, *W*. The upper scale shows the temperature increase due to optical excitation of the Al film. Source data

To validate the suggested approach, we present the results of an experiment in which we use polariton probing for the detection of a strain pulse with an amplitude as low as *η*_0_ ≈ 2 × 10^−9^, corresponding to a displacement amplitude of *u*_0_ < 10^−16^ m and thus reaching the attometre (am) range. The scheme of our experiment performed at the bath temperature *T*_0_ = 10 K is shown in Fig. 2a. The medium hosting the polariton resonance is a GaAs/AlGaAs multiple quantum well (MQW) layer grown on a GaAs substrate (Methods). The permittivity of the polaritonic layer is described by the resonant dielectric function^49^:

3 $$\varepsilon ={\varepsilon }_{{\mathrm{b}}}\left(1+\frac{{E}_{{{\mathrm{LT}}}}}{{E}_{0}-E-i\varGamma }\right),$$

where *ε*_b_ = 11.2 is the background dielectric constant, *E*_LT_ = 0.13 meV is the longitudinal–transverse splitting, *E*_0_ = 1.5307 eV is the energy of the exciton resonance in the quantum wells, *i* is the imaginary unit and *Γ* = 0.7 meV is the polariton decay rate (the procedure of obtaining the parameters of the polariton resonance is described in Methods).

As a stress source, we use optical heating of the electron gas in a metal. Femtosecond pump laser pulses excite an Al film with thickness *ξ* = 100 nm deposited on the surface opposite the polaritonic MQW layer. The optically excited hot electrons pass their energy to the lattice in less than 1 ps and generate thermal stress, resulting in thermal expansion of the Al film and injection of an acoustic pulse into GaAs (ref. ^42^). The acoustic pulse propagates through the GaAs substrate with the longitudinal sound velocity *v* = 4,800 m s^−1^, finally reaching the polaritonic layer. The blue line in Fig. 2b shows the simulated temporal profile of the displacement *u*(*t* − *z*/*v*) within the pulse, whose amplitude is proportional to the optical pump pulse fluence *W* (ref. ^50^). The main part of this displacement profile has a Gaussian shape. The small satellite at later times is the result of the acoustic reflection at the Al/GaAs interface. The amplitude *u*_0_ = 1.0 × 10^−16^ m corresponds to the pump fluence *W* = 30 nJ cm^−2^ (Methods). The red line in Fig. 2b gives the calculated strain *η*(*t* – *z*/*v*) = d*u*/d*z* with amplitude *η*_0_ = 2.7 × 10^−9^ for the same pump fluence *W*.

In the experiments, we measure the transient reflectivity Δ*R*(*t*) for probe pulses with wavelength *λ*_0_ = 810 nm (corresponding to *E* = *E*_0_). Examples of measured transient signals are shown in Fig. 2c for three values of the pump fluence *W*, where an oscillatory behaviour of Δ*R*(*t*) is clearly seen. All signals have a ‘turning point’ at *t* = 0 due to 100% reflection of the acoustic wave from the free front surface of the sample (*t* = 0 corresponds to the time moment when the centre of the main acoustic pulse hits the front surface). The red line corresponds to the fluence *W* = 30 nJ cm^–2^ and the related displacement amplitude *u*_0_ = 100 am. At this value of *W*, the oscillations in Δ*R*(*t*) clearly stand out from the noise. The minimum value of *W* for which the oscillations can be distinguished in our experiment is *W* = 10 nJ cm^−2^ with the corresponding *u*_0_ = 30 am. The black line at the bottom of Fig. 2c shows the signal calculated using the transfer matrix method (Methods). We see good agreement between the measured and calculated temporal shapes of Δ*R*(*t*): the same sign at the turning point at *t* = 0 and a slight asymmetry of the left (*t* < 0) and right (*t* > 0) parts (that is, the oscillation at *t* > 0 lasts longer) due to the finite duration of the acoustic pulse. The amplitude of the oscillations in the calculated signal is slightly larger than that of the measured one (compare the red and black curves), which can be explained by not taking into account the finite spectral width of the probe beam in the calculations. Figure 2d shows the normalized fast Fourier transforms of the experimental and calculated Δ*R*(*t*) values. Both spectra consist of a spectral line with a maximum at a frequency *f* = 41 GHz, which corresponds to the Brillouin frequency *f*_B_ for the used probe photon energy and the corresponding permittivity of the polaritonic layer. The spectral widths of both lines (full-widths at half-maxima) are ~4 GHz, determined by the penetration depth of the probe beam in the polaritonic layer.

## Dynamical range of polariton probing

Next we show that polariton probing of acoustic pulses offers a wide dynamical range across which the amplitude of the measured transient reflectivity signal grows linearly with an increase of the displacement and dynamical strain. The symbols in Fig. 2e represent the measured dependence of the amplitude *A* (Methods) of the transient reflectivity signal on pump fluence *W*. The dependence remains linear in a range of pump fluences covering four orders of magnitude: from 10 nJ cm^–2^ to 100 µJ cm^–2^. We can estimate the thermal stress *σ*_Al_ generated as a result of the optically induced instantaneous lattice temperature increase Δ*T* of the Al film in the following way for this range of fluences^51^:

4 $${\sigma }_{{{\mathrm{Al}}}}\left(z\right)={\int }_{{T}_{0}}^{{T}_{0}+\Delta T(z)}\gamma {C}_{V}\left(T\,\right){{\mathrm{d}}T},$$

where *γ* ≈ 1.9 is the Grüneisen parameter of Al (ref. ^52^), and *C*_*V*_ is the specific heat per unit volume *V*. For a constant value Δ*T* across the whole thickness of the metal film (its calculated values for the range of *W* used are shown by the upper scale in Fig. 2e; details are in Methods), we get

5 $${\bar{\sigma }}_{{\rm{Al}}}=\frac{\gamma W\left(1-{R}_{{\rm{Al}}}\right)}{\xi },$$

where *R*_Al_ = 0.85 is the reflection coefficient of the pump light from the Al film with thickness *ξ* = 100 nm. The calculated dependence of ${\bar{\sigma }}_{{\rm{Al}}}$ on *W* is shown in Fig. 2e by the blue line. The relation of the transient reflectivity amplitude *A* and the optically generated stress ${\bar{\sigma }}_{{\rm{Al}}}$ in the Al film reads $A=a{\bar{\sigma }}_{{\rm{Al}}}$ with *a* = 3 GPa^−1^, independent of *W* and Δ*T*. The experimental dependence of the signal amplitude and the calculated dependence of the optically generated stress on *W* perfectly match the linear dependence given by equation (5). This shows the reliability of polariton detection for obtaining the precise value of the generated stress in a range covering at least four orders of magnitude.

## Polariton probing sensitivity limits

Finally, we define the strain sensitivity of polariton probing *α*, which relates the strain amplitude carried by the acoustic pulse and the amplitude of the transient reflectivity signal such that *A* = *αη*_0_, achieved in our experiment. In general, *α* depends on the temporal evolution of the strain in the acoustic pulse (red curve in Fig. 2b) and, correspondingly, its spectral density at *f* = *f*_B_. The inset in Fig. 2b shows the calculated fast Fourier transform spectrum $\widetilde{\eta }(f)$ for the *η*(*t*) demonstrated in the main panel of Fig. 2b. This spectrum has the maximum around *f* = *f*_B_, and for these optimal conditions, we get *α* ≈ 2 × 10^3^. This sensitivity is high enough to detect the picosecond atomic displacement, delivered from the Al film to the MQW structure, with an amplitude below 100 am. It is four orders of magnitude less than the averaged amplitude of thermal atomic motion, 〈*u*_*T*_〉, which is ~1 pm at the bath temperature *T*_0_ = 10 K ($\left\langle {u}_{T}\right\rangle \approx \sqrt{{k}_{{\mathrm{B}}}{T}_{0}/{C}_{11}{a}_{l}}$, where *k*_B_ = 1.38 × 10^–23^ J K^–1^ is the Boltzmann constant, and *C*_11_ = 116.3 (120) GPa and *a*_*l*_ = 4.05 (5.63) Å are the elastic and lattice constants of Al (GaAs), respectively). However, the highest sensitivity for both strain and displacement could be obtained for a harmonic acoustic wave with *f* = *f*_B_. For such a wave, we estimate the sensitivity in the used polaritonic layer as *α* ≈ 1.5 × 10^4^, which is enough to detect a harmonic acoustic signal with a displacement of only ~1 am. The estimated differential reflectivity signal amplitude for such an acoustic wave of *A* ≈ 10^−6^ is detectable with the optical set-up used in our experiment. By comparison, if we substitute the polaritonic layer with a transparent medium without polariton resonance, for example, SiO_2_ or Al_2_O_3_, in which the optical detection is averaged over the whole coherence length of the laser pulses, the strain sensitivity for the corresponding harmonic acoustic wave will be ${\alpha }_{{\rm{Si}}{{\rm{O}}}_{2}}\approx {3.0\times 10}^{3}$ or ${\alpha }_{{{\rm{Al}}}_{2}{{\rm{O}}}_{3}}\approx {4.0\times 10}^{2}$, respectively. The numbers and comparison given above unambiguously prove the unique sensitivity of the suggested polariton probing.

## Conclusions

In conclusion, we have shown that polariton probing of high-frequency acoustic pulses enables monitoring of ultimately small lattice displacements and strains. We have validated the method by detecting the optically induced thermal expansion of a 100-nm-thick Al film due to a remarkably low-temperature increase of 0.2 K. The corresponding expansion by 100 am and the related strain of 10^−9^ were confidently detected. The amplitude variation of the measured transient reflectivity signal with pump fluence remains linear in a range covering more than four orders of magnitude, in accordance with the constant value of the Grüneisen parameter of Al in the corresponding heating range. This confirms the wide dynamical range of polariton probing, which may be applied to new materials for studying complex dependences of the Grüneisen parameter on temperature and clarifying mechanisms of thermally induced^53^, pressure-induced^54^ and light-induced^55^ phase transitions. Its sensitivity combined with the method of tomographic reconstruction of high-frequency acoustic pulses^56^ can provide even more detailed information about the studied ultrafast process. Such sensitive detection also opens the possibility for operation with high-frequency acoustic waves generated by pulsed lasers with pulse repetition rates equal to an integer divisor of the Brillouin frequency, commercially available nowadays^57^. The method also corroborates the proposal of polaritonic detection of single high-frequency phonon quanta^58^. And, the effect of a high-frequency acoustic pulse on the polariton spectrum allows for probing the spatiotemporal profile of the internal optical field inside a polaritonic structure^59^.

## Methods

### Sample fabrication

The studied structure consists of 30 pairs of Ga_0.67_Al_0.33_As and GaAs layers with thicknesses of 8 nm and 17.5 nm, respectively, covered by a single 8 nm layer of Ga_0.67_Al_0.33_As and a 5 nm layer of GaAs (ref. ^59^). The total thickness of the polaritonic layer is *l*_MQW_ = 778 nm. It was grown by molecular beam epitaxy on a semi-insulating GaAs(001) substrate on top of a 100 nm GaAs buffer. For the experiments, the substrate was polished down to 105 µm thickness, and an Al film of 100 nm thickness was deposited on its back side by magnetron sputtering.

### Measurements

The sample was mounted on a metallic cold finger in the vacuum chamber of a flow helium cryostat. The parameters of the polariton resonance, that is, spectral position and width, were obtained from a reflectivity spectrum measured using a halogen lamp and a 0.5 m single monochromator with a charge-coupled device (CCD) camera. The time-resolved measurements were carried out in a conventional pump–probe scheme^59^. The laser source for the transient reflectivity measurements was a tuneable Ti:sapphire laser oscillator with 76 MHz pulse repetition rate and 120 fs pulse duration. The photon energy was tuned to the spectral position of the polariton resonance, *ħω* = 1.5307 eV (wavelength *λ* = 810 nm). The laser beam was split into the pump and probe beams by a 50:50 beam-splitter. The pump beam was focused on the surface of the Al film to a spot of 50 µm diameter (full-width of the Gaussian intensity distribution at the level 1/*e*^2^ of the maximum intensity, where *e* is the base of the natural logarithm function). The probe beam was retarded by a fixed mechanical delay line by 20 ns (travelling time of the acoustic pulse through the GaAs substrate) and passed through a tuneable double liquid crystal filter (VariSpec) with a transmission band of 0.8 meV spectral width centred at the laser photon energy. The probe beam was focused on the front surface of the polaritonic layer to a spot of 40 µm diameter, opposite to the pump spot. The intensity of the probe pulse reflected from the sample was measured by a Si photoreceiver with 500 kHz bandwidth. Temporal resolution was provided by an automatized variable delay line installed in the optical path of the pump beam. The signal-to-noise ratio was improved by lock-in detection exploiting a 10 MHz lock-in amplifier synchronized with a mechanical chopper, which interrupted the pump beam at a frequency of 5.5 kHz. The Matlab R2019a software was used to control the data acquisition.

### Parameters of the measured signals

The main parameters of the measured signals, such as amplitude *A* and frequency *f*, were obtained by fitting the signals with the function $\frac{\Delta R}{{R}_{0}}=A(W\,)\sin \left(2\uppi {ft}-{\varphi }_{-(+)}\right)\exp (-\frac{t}{\varsigma \tau })$, where *t* = 0 corresponds to the moment when the centre of the main part of the acoustic pulse is reflected at the open front surface of the polaritonic layer, *φ*_−(+)_ is the transient signal phase at *t* < 0 (*t* > 0), *τ* is the decay time determined by the penetration depth of the probe beam into the polaritonic layer and *ς* = −1 or 1 for *t* < 0 or *t* > 0, respectively. The frequency, phase and decay time do not depend on *W* in the entire range of pump fluences used. All fitting of the measured signals as well as fast Fourier transforms were performed with the OriginPro 2023 software.

### Calculation of the optically induced heating of the Al film

For the calculation of the temperature increase induced by the femtosecond pump pulse, we modelled the temperature dependence of the heat capacity in the experimental temperature range using data from ref. ^60^ as

6 $$C_{V}\left(T\,\right)=\rho\left(6.6\times {10}^{-2}T+{10}^{-3}{T}^{\,3}\right),$$

where *ρ* = 2.7 × 10^3^ kg m^−3^ is the Al mass density. For the electron–phonon coupling constant we assumed *g* = 5 × 10^17^ W m^−3^ K^−^^1^ (ref. ^51^), and the estimated time of electron–lattice thermalization is ~0.1 ps. The electron diffusion coefficient for Al at liquid helium temperatures is ~0.15 m^2^ s^–1^ (ref. ^61^), which results in a thermal diffusion time through the 100 nm Al film of ~0.3 ps. Then we may assume that a uniform temperature distribution is established across the Al film thickness almost instantly after the optical pump excitation. In this case, the temperature increase for the pump fluence, *W*, can be calculated as follows:

7 $$\Delta T=\frac{\left(1-{R}_{{{\mathrm{Al}}}}\right)W}{C_{V}\xi },$$

where *R*_Al_ = 0.85 is the Al reflectance and *ξ* = 100 nm is the film thickness.

### Modelling the transient reflectivity signals

For numerical modelling of the transient reflectivity signals we used the transfer matrix formalism. We consider the polaritonic layer as an effective medium with space-dependent and time-dependent permittivity, in accordance with the propagating strain pulse *η*(*z*,*t*). We used the dispersion given by equation (3) with the space-dependent and time-dependent polariton energy, *E*_0_ + Δ*E*_0_(*z*,*t*), where Δ*E*_0_(*z*,*t*) = *Ξη*(*z*,*t*) is the resonance energy shift induced by the strain *η*(*z*,*t*). The deformation potential of GaAs is *Ξ* = –10 eV (ref. ^62^). For calculating the spatiotemporal evolution of the strain, we modelled the acoustic pulse by the Gaussian-like displacement profile shown in Fig. 2b. The sound velocity in the polaritonic layer, *v*_MQW_ = 5.04 km s^−1^, was obtained from the experimentally measured Brillouin frequency. The absolute values of *u*_0_ were obtained by normalizing the data from ref. ^50^. We checked that in the entire range of pump fluences, the amplitude of the calculated transient reflectivity signal remains linearly dependent on *W*. To calculate the strain sensitivity for the harmonic acoustic wave, we substitute the Gaussian acoustic pulse with a semi-infinite acoustic wave with *f* = *f*_B_. The reflection at the open surface was not taken into account. To calculate the corresponding sensitivity for transparent SiO_2_ and Al_2_O_3_, we used the following dielectric functions and photoelastic coefficients: ${\varepsilon }_{{\rm{Si}}{{\rm{O}}}_{2}}=2.1$, ${p}_{12}^{{\rm{Si}}{{\rm{O}}}_{2}}=0.3$, ${\varepsilon}_{{\rm{Al}}_{2}{\rm{O}}_{3}}=3.1$ and ${p}_{13}^{{{\rm{Al}}}_{2}{{\rm{O}}}_{3}}=0.02$ (ref. ^63^). The wavelength and frequency of the acoustic wave were adjusted to fulfil the Brillouin selection rule accordingly. The modelling of the transient reflectivity signals was performed using the Matlab R2024a software.

### Reporting summary

Further information on research design is available in the Nature Portfolio Reporting Summary linked to this article.

## Online content

Any methods, additional references, Nature Portfolio reporting summaries, source data, extended data, supplementary information, acknowledgements, peer review information; details of author contributions and competing interests; and statements of data and code availability are available at 10.1038/s41563-025-02229-3.

## Supplementary information

Reporting Summary

## Source data

Source Data Fig. 1 Simulated curves and a colour map presented in Fig. 1a–f. Each panel is presented in a separate sheet with the corresponding name.

Source Data Fig. 2 Calculated strain and displacement temporal profiles (Fig. 2b); normalized experimental and simulated signals (Fig. 2c); the fast Fourier transform of the experimental and simulated signals for the pump fluence 600 nJ cm^–2^ (Fig. 2d); and experimental and calculated pump fluence dependences of the signal amplitude and thermal stress, respectively (Fig. 2e). The source data for each panel are presented in a separate sheet with the corresponding name.

## Data Availability

All experimental data that support the findings of this study are available via Zenodo at 10.5281/zenodo.15020024 (ref. ^64^). Source data are provided with this paper.
