# Supplementary material for: Polariton probing of attometre displacement and nanoscale strain in ultrashort acoustic pulses
Source: Nat Mater. 2025 May 15;24(8):1209–14. doi: 10.1038/s41563-025-02229-3 (PMC12310516; doi:10.1038/s41563-025-02229-3)
Supplement: Supplementary file 1 — Reporting Summary [file 41563_2025_2229_MOESM1_ESM.pdf]

nature portfolio

Corresponding author(s): Alexey V. Scherbakov

Last updated by author(s): Mar 23, 2025

Reporting Summary

Nature Portfolio wishes to improve the reproducibility of the work that we publish. This form provides structure for consistency and transparency in reporting. For further information on Nature Portfolio policies, see our Editorial Policies and the Editorial Policy Checklist.

Statistics

For all statistical analyses, confirm that the following items are present in the figure legend, table legend, main text, or Methods section.

n/a	Confirmed	
☒	☐	The exact sample size (*n*) for each experimental group/condition, given as a discrete number and unit of measurement
☐	☒	A statement on whether measurements were taken from distinct samples or whether the same sample was measured repeatedly
☒	☐	The statistical test(s) used AND whether they are one- or two-sided<br>*Only common tests should be described solely by name; describe more complex techniques in the Methods section.*
☒	☐	A description of all covariates tested
☒	☐	A description of any assumptions or corrections, such as tests of normality and adjustment for multiple comparisons
☒	☐	A full description of the statistical parameters including central tendency (e.g. means) or other basic estimates (e.g. regression coefficient) AND variation (e.g. standard deviation) or associated estimates of uncertainty (e.g. confidence intervals)
☒	☐	For null hypothesis testing, the test statistic (e.g. *F*, *t*, *r*) with confidence intervals, effect sizes, degrees of freedom and *P* value noted<br>*Give P values as exact values whenever suitable.*
☒	☐	For Bayesian analysis, information on the choice of priors and Markov chain Monte Carlo settings
☒	☐	For hierarchical and complex designs, identification of the appropriate level for tests and full reporting of outcomes
☒	☐	Estimates of effect sizes (e.g. Cohen's *d*, Pearson's *r*), indicating how they were calculated

Our web collection on statistics for biologists contains articles on many of the points above.

Software and code

Policy information about availability of computer code

Data collection	The data was collected using the analog to digital input of the Zurich Instruments Lock-In HF2LI and following transfer of the demodulated signal to a 16-bit transient recorder M2i.4911-exp by Spectrum Instrumentation. The data acquisition process was controlled by Mathlab R2019a software.
Data analysis	Figure preparation including the fast Fouriertransformation was done using the OriginPro 2023. The simulated transient signal was calculated using Mathlab R2024a.

For manuscripts utilizing custom algorithms or software that are central to the research but not yet described in published literature, software must be made available to editors and reviewers. We strongly encourage code deposition in a community repository (e.g. GitHub). See the Nature Portfolio guidelines for submitting code & software for further information.

Data

Policy information about availability of data

All manuscripts must include a data availability statement. This statement should provide the following information, where applicable:

- Accession codes, unique identifiers, or web links for publicly available datasets
- A description of any restrictions on data availability
- For clinical datasets or third party data, please ensure that the statement adheres to our policy

All experimental data that support the findings of this study have been deposited in the Zenodo database under https://doi.org/10.5281/zenodo.15020024.

Research involving human participants, their data, or biological material

Policy information about studies with human participants or human data. See also policy information about sex, gender (identity/presentation), and sexual orientation and race, ethnicity and racism.

Reporting on sex and gender	does not apply
Reporting on race, ethnicity, or other socially relevant groupings	does not apply
Population characteristics	does not apply
Recruitment	does not apply
Ethics oversight	does not apply

Note that full information on the approval of the study protocol must also be provided in the manuscript.

Field-specific reporting

Please select the one below that is the best fit for your research. If you are not sure, read the appropriate sections before making your selection.

☒ Life sciences ☐ Behavioural & social sciences ☐ Ecological, evolutionary & environmental sciences

For a reference copy of the document with all sections, see nature.com/documents/nr-reporting-summary-flat.pdf

Life sciences study design

All studies must disclose on these points even when the disclosure is negative.

Sample size	The physical size of the examined sample was 5x5x0.1 mm^3 (WxHxD), so the area of the back and front surfaces illuminated by the pump and probe laser beams was much larger than the laser spot size (~10 µm). It allowed us to randomly select a point on the sample surface each experimental day (20 days in total).
Data exclusions	No data was excluded from the analysis
Replication	Experimental studies were carried out during 15 experimental days. On each experimental day, the experimental setup was re-adjusted with a random selection of the region of the investigated structure. To confirm the signal reproducibility, the control measurements under certain experimental conditions (wavelength and fluence of the pump and probe lasers, temperature) were performed. The signals obtained in this way show excellent reproducibility.
Randomization	In addition to a randomly selected area on the sample, the dependencies of signal parameters on the pump fluence, its value was varied non-monotonically, but in random up /down steps to avoid affectation of the dependence by possible accumulating changes in experimental conditions during the experimental day.
Blinding	The design of the experiment and polariton structure were adjusted to demonstrate the ultimate sensitivity of polaritonic detection of dynamical strain and the members of the research group were informed about the goal of the study. Nevertheless, theoretical calculations of the parameters of the acoustic pulses and their effect on the polariton spectrum were based on data from the cited articles, in the absence of any information about the experimental signals obtained. Similarly, the analysis of the experimental signals was performed without information about the results of the theoretical calculation.

Reporting for specific materials, systems and methods

We require information from authors about some types of materials, experimental systems and methods used in many studies. Here, indicate whether each material, system or method listed is relevant to your study. If you are not sure if a list item applies to your research, read the appropriate section before selecting a response.

Materials & experimental systems

n/a	Involved in the study
☒	☐ Antibodies
☒	☐ Eukaryotic cell lines
☒	☐ Palaeontology and archaeology
☒	☐ Animals and other organisms
☒	☐ Clinical data
☒	☐ Dual use research of concern
☒	☐ Plants

Methods

n/a	Involved in the study
☒	☐ ChIP-seq
☒	☐ Flow cytometry
☒	☐ MRI-based neuroimaging

Plants

Seed stocks	does not apply
Novel plant genotypes	does not apply
Authentication	does not apply
